# Supplementary material for: LTA4H extensively associates with mRNAs and lncRNAs indicative of its novel regulatory targets
Source: PeerJ. 2023 Mar 10;11:e14875. doi: 10.7717/peerj.14875 (PMC10010175; doi:10.7717/peerj.14875)
Supplement: File S2 [file peerj-11-14875-s005.zip › Raw data files/Motif_results/cims/LTA4H_IP_1_vs_LTA4H_input_1/homerResults/motif10.info.html]

Motif 10

## Information for 13-TGRTGRTGAT (Motif 10)

  
Reverse Opposite:
